# Direct conversion of human fibroblasts into dopaminergic neuron-like cells using small molecules and protein factors

**DOI:** 10.1186/s40779-020-00284-2

**Published:** 2020-11-01

**Authors:** Hua Qin, An-Dong Zhao, Meng-Li Sun, Kui Ma, Xiao-Bing Fu

**Affiliations:** 1 Research Center for Tissue Repair and Regeneration affiliated to the Medical Innovation Research Division and 4th Medical Center, PLA General Hospital and PLA Medical College, 28 Fu Xing Road, Haidian District, Beijing, 100853 China; 2 Tianjin Medical University, Tianjin, 300070 China; 3 PLA Key Laboratory of Tissue Repair and Regenerative Medicine and Beijing Key Research Laboratory of Skin Injury, Repair and Regeneration, Beijing, 100048 China; 4 Research Unit of Trauma Care, Tissue Repair and Regeneration, Chinese Academy of Medical Sciences, 2019RU051, Beijing, 100048 China

**Keywords:** Human fibroblasts, Dopaminergic neurons, Parkinson’s disease, Small molecules, Reprogramming, Transdifferentiation

## Abstract

**Background:**

Generation of neurons is essential in cell replacement therapy for neurodegenerative disorders like Parkinson’s disease. Several studies have reported the generation of dopaminergic (DA) neurons from mouse and human fibroblasts by ectopic expression of transcription factors, in which genetic manipulation is associated with potential risks.

**Methods:**

The small molecules and protein factors were selected based on their function to directly induce human fetal lung IMR-90 fibroblasts into DA neuron-like cells. Microscopical, immunocytochemical, and RT-qPCR analyses were used to characterize the morphology, phenotype, and gene expression features of the induced cells. The whole-cell patch-clamp recordings were exploited to measure the electrophysiological properties.

**Results:**

Human IMR-90 fibroblasts were rapidly converted into DA neuron-like cells after the chemical induction using small molecules and protein factors, with a yield of approximately 95% positive TUJ1-positive cells. The induced DA neuron-like cells were immunopositive for pan-neuronal markers MAP2, NEUN, and Synapsin 1 and DA markers TH, DDC, DAT, and NURR1. The chemical induction process did not involve a neural progenitor/stem cell intermediate stage. The induced neurons could fire single action potentials, which reflected partially the electrophysiological properties of neurons.

**Conclusion:**

We developed a chemical cocktail of small molecules and protein factors to convert human fibroblasts into DA neuron-like cells without passing through a neural progenitor/stem cell intermediate stage. The induced DA neuron-like cells from human fibroblasts might provide a cellular source for cell-based therapy of Parkinson’s disease in the future.

## Background

Regeneration of cell types that are lost or damaged is essential for regenerative medicine [1]. Advances in understanding the mechanisms of embryonic development and cell differentiation have provided helpful guidance on how to control cell fate. One theory is that the expression of specific transcription factors is involved in the selection of cell fates and their subsequent differentiation [2–4]. Based on the theory, ectopic expression of transcription factors has been widely used to reprogram or transdifferentiate or convert somatic cells into pluripotent stem cells, multipotent stem cells, and other somatic cells [5]. However, the transcription factor-based strategies are faced with certain safety concerns in terms of future clinical use because genome integration of ectopic genes is possible to occur. Accordingly, researchers try to find an alternative route to replace the transcription factors. In addition to transcription factors, regulation of the signaling pathways using small molecules has been demonstrated to successfully induce cell fate conversion in recent years [6–9]. Reprogramming the cells utilizing small molecules instead of transcription factors excels in several aspects. First, small molecules are permeable and thus easily manipulated. Second, the effects of small molecules are reversible and conveniently controlled by adjusting concentrations and combinations. In addition, it’s cost-effective since small molecules could be synthesized. Therefore, the small molecule-based strategy for cell fate conversion may be potentially translated into clinical therapy.

Parkinson’s disease is one of the most common neurodegenerative disorder characterized by degeneration and loss of dopaminergic (DA) neurons in the substantial nigra pars compacta [10]. Cell replacement therapy holds a promising strategy for this disease. Several studies have generated DA neurons from mouse and human fibroblasts by ectopic expression of different combinations of transcription factors via lentil virus delivery [11–16]. However, potential risks associated with genetic manipulations may limit its future applications.

Based on the knowledge of neuronal development and DA neuron differentiation, we developed a chemical method to convert human fibroblasts into DA neuron-like cells using small molecules and proteins. The induced human DA neuron-like cells (ihDAs) exhibited neuronal morphology, expressed pan-neural markers and DA makers, and possessed electrophysiological properties. Further works are required to evaluate the in vivo function of ihDAs after transplantation, which is critical for developing cell replacement therapy to treat Parkinson’s disease in the future.

## Methods

### Cell culture

Human fetal lung IMR-90 fibroblasts were purchased from Stem Cell Bank, Chinese Academy of Sciences. IMR-90 fibroblasts were maintained in the routine culture medium consisting of Dulbecco’s modified eagle medium (DMEM, Invitrogen) supplemented with 10% fetal bovine serum (FBS, Invitrogen), 2 mmol/L GlutaMAX (Invitrogen), 1% penicillin/streptomycin (Gibco) at 37 °C with 5% CO_2_.

### Chemical induction of IMR-90 fibroblasts into ihDAs

**Fig. 1:**
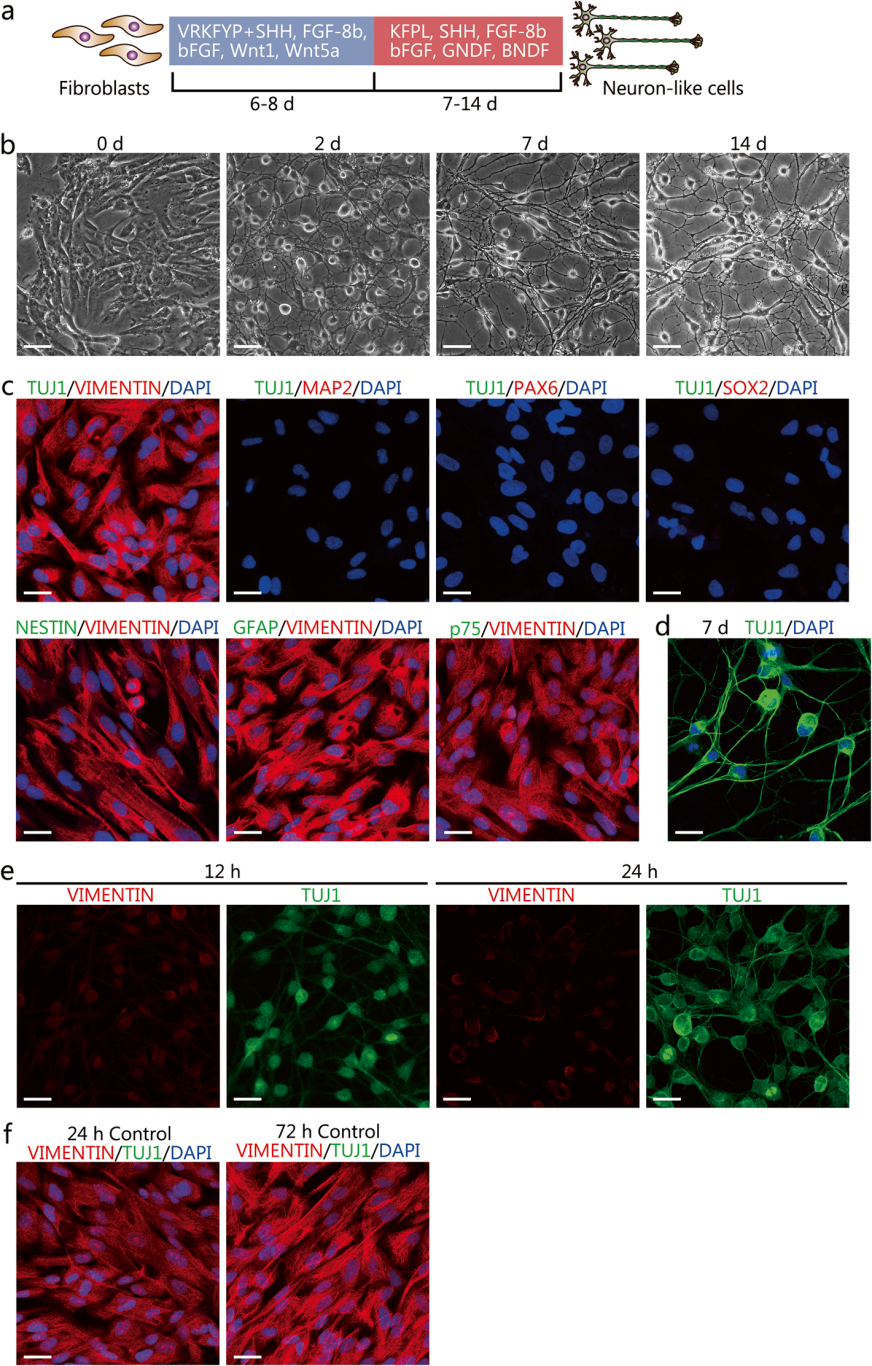
Chemical induction of human lung fetal IMR-90 fibroblasts into neuron-like cells. **a** A schematic diagram describing the chemical induction procedure. V, VPA; R, Repsox; K, kenpaullone; F, forskolin; Y, Y-27632; P, purmorphamine; L, L-ascorbic acid. **b** Representative microscopy images depicting the morphological changes after chemical induction. Scale bars=100 μm. **c** Characterization of the cultured IMR-90 fibroblasts by immunostaining assay for the fibroblast marker VIMENTIN, neural markers Class III β-tubulin 1 (TUJ1) and microtubule-associated protein 2 (MAP2), neural progenitor/stem cell markers PAX6, SOX2, and NESTIN, glial cell marker GFAP, and neural crest cell marker p75. Scale bars=25 μm. **d** Expression of TUJ1 after 7 days of chemical inductions. Scale bar = 25 μm. **e** Dynamic changes of VIMENTIN and TUJ1 in fibroblasts at early 12 h and 24 h of chemical induction. Scale bars=25 μm. **f** Dynamic changes of VIMENTIN and TUJ1 at early 24 h and 72 h in the control medium with small molecules and protein factors. Scale bars=25 μm

IMR-90 fibroblasts were seeded onto 6-well plates or glass coverslips coated with the matrigel (Corning) diluted in DMEM (1:40). After the cell confluence reached about 70–90%, the culture medium was replaced with the neuronal induction medium consisting of neurobasal medium (Gibco) supplemented with 0.5% N_2_, 1% B27, 2 mmol/L GlutaMAX, 1% penicillin/streptomycin along with small molecules and growth factors. The small molecules and growth factors and their concentrations used in the study were: 0.1 mmol/L valproic acid (VPA, MedChem Express, #HY-10585), 2 μmol/L Repsox (MedChem Express, #HY-13012), 3 μmol/L kenpaullone (MedChem Express, #HY-12302), 5 μmol/L forskolin (Selleck, #S2449), 5 μmol/L Y-27632 (Selleck, #S1049), and 2 μmol/L purmorphamine (MedChem Express, #HY-15108), 100 ng/ml human Sonic Hedgehog (SHH, PeproTech, #100–45), 100 ng/ml fibroblast growth factor-8b (FGF-8b, PeproTech, #100–25), 20 ng/ml basic fibroblast growth factor (bFGF, PeproTech, #100-18B), 50 ng/ml Wnt1 (PeproTech, #120–17), and 50 ng/ml Wnt5a (R&D systems, #645-WN). After 6–8 days of chemical induction, the induction medium was replaced with the neuronal maturation medium with 5 μmol/L forskolin, 1 μmol/L kenpaullone, 0.2 mmol/L L-ascorbic acid (MedChem Express, #HY-B0166A), 100 ng/ml SHH, 100 ng/ml FGF-8b, 20 ng/ml bFGF, 10 ng/ml brain-derived neurotrophic factor (BDNF), 10 ng/ml glial cell-derived neurotrophic factor (GDNF). Cells were cultured for additional 7–14 days, with fresh maturation medium replaced every 2 days. In the control group, IMR-90 fibroblasts were cultured in the medium consisting of neurobasal medium supplemented with 0.5% N_2_, 1% B27, 2 mmol/L GlutaMAX and 1% penicillin/streptomycin with 1% DMSO, without small molecules and protein factors. The induction strategy was illustrated in Fig. 1a.

### BrdU incorporation assay

IMR-90 fibroblasts were treated with 5-bromo-2-deoxyuridine (BrdU, Millipore) at a concentration of 10 μmol/L for 2 h before immunocytochemical analysis during the chemical induction process.

After labeling cells, the labeling medium containing BrdU was removed. Cells were washed twice with phosphate-buffered saline (PBS), followed by fixation with 4% formaldehyde (PFA) at room temperature (RT) for 15 min. After three washes with PBS, cells were incubated in Triton X-100 permeabilization buffer for 20 min at RT. After removal of the permeabilization buffer, cells were incubated in 1 N HCl at 37 °C for 30 min. Then, the HCl solution was removed, and cells were neutralized with 0.1 mol/L sodium borate buffer (pH 8.5) for 10 min at RT. After three washes with Triton X-100 permeabilization buffer for 3 min each time, cells were blocked with 5% donkey serum, 1% bovine serum albumin, 0.3% Triton X-100 in PBS for 30 min at RT. Then the primary mouse anti-BrdU (1:50, 2750, Millipore) antibody and rabbit anti-TUJ1 (1:500, T2200, Sigma) were used for incubation at 4 °C overnight. After three washes with PBS, cells were incubated with the Alexa 488 and Alexa 594-conjugated secondary antibodies for 1 h at RT. Images were captured using a Leica Sp8 confocal microscope.

### Immunocytochemical analysis

For immunostaining analysis, cells were fixed with 4% PFA for 10 min at RT, followed by three washes with PBS, and blocked with 5% donkey serum, 1% bovine serum albumin, and 0.3% Triton X-100 in PBS for 30 min at RT. Cells were then incubated with primary antibodies at 4 °C overnight and washed 3 times with PBS, followed by incubation with appropriate fluorescent probe-conjugated secondary antibodies for 1 h at RT. Cell nuclei were counterstained with DAPI. Images were obtained with a Leica Sp8 confocal microscope. The following primary antibodies were used: rabbit anti-VIMENTIN (1:200, 5741, Cell Signaling Technology), mouse anti-TUJ1 (1:500, 801,201, Covance), rabbit anti-TUJ1(1:500, T2200, Sigma), rabbit anti-MAP2 (1:500, AB5622, Millipore), rabbit anti-SYN (1:500, AB1543, Millipore), mouse anti-NEUN (1:200, MAB377, Millipore), rabbit anti-TH (1:200, AB152, Millipore), mouse anti-TH (1:200, Sigma-Aldrich), rabbit ani-GABA (1:500, A2052, Sigma), rabbit anti-vGlut1 (1:1000, 48–2400, Invitrogen), goat anti-CHAT (1:200, AB144P, Millipore), rabbit Anti-DAT (1:1000, MAB369, Millipore), rabbit anti-DDC (1:200, AB1569, Millipore), mouse anti-NURR1 (1:100, sc-376,984, Santa Cruz), rabbit anti-Ki67 (1:500, ab15580, Abcam), rabbit anti-PAX6 (1:500, 901,301, Biolegend), rabbit anti-SOX2 (1:200, AB5603, Millipore), mouse anti-NESTIN (1:200, MAB53026, Millipore), mouse anti-p75 (1:100, sc-13,577, Santa Cruz), and mouse anti-GFAP (1:100, sc-33,673, Santa Cruz). The Alexa 488 and Alexa 594-conjugated secondary antibodies were obtained from Jackson Immunoresearch Laboratories.

### Chemical induction efficiency

The chemical conversion efficiency was calculated as previously described [17–19]. Briefly, 10 randomly selected visual fields (× 20) were used to determine the cell numbers. The total number of TUJ1^+^ cells and TUJ1^+^TH^+^ cells displaying neuronal morphology were counted. The neuronal conversion efficiency was calculated as the percentages of TUJ1^+^ cells relative to the total DAPI^+^ cells. The conversion efficiency and purity of dopaminergic neuron-like cells were calculated as the percentage of TUJ1^+^TH^+^ cells relative to the total DAPI^+^ cells or the total TUJ1^+^ cells.

### Measuring cell proliferation after chemical induction

To evaluate the changes of proliferation state after chemical induction, we calculated the percentages of Ki67^+^, TUJ1^+^Ki67^+^ and TUJ1^+^BrdU^+^ cells during the early 72 h after induction. For Ki67 and TUJ1 immunostaining, 10 randomly selected × 20 visual fields from each sample were used to determine the cell numbers by counting the total cell number of Ki67^+^ cells and TUJ1^+^Ki67^+^ cells, respectively. The percentage of Ki67^+^ cells relative to the total DAPI^+^ cells was calculated from 0 h to 72 h after chemical induction, and the percentage of TUJ1^+^Ki67^+^ cells in the TUJ1^+^ cells at 12, 24, 48, and 72 h after induction was calculated. For BrdU and TUJ1 immunostaining, the total cell number of TUJ1^+^BrdU^+^ cells and TUJ1^+^ cells were counted at 12, 24, 48, and 72 h after chemical induction. The percentage of TUJ1^+^BrdU^+^ cells relative to TUJ1^+^ cells was calculated.

### Quantitative real-time PCR

Total RNA was extracted from cell samples with Trizol (Invitrogen). Isolated RNA was reverse-transcribed with the PrimeScript RT reagent kit with a gDNA eraser (TaKaRa, # RR047A). Quantitative real-time PCR was performed by using SYBR Premix Ex Taq II (TaKaRa) in a 7300 Real-time PCR system (Applied Biosystems). The relative expression levels of the target genes were normalized to that of the internal control (GAPDH). The primer sequences are listed in Table 1.

**Table 1:** Primer lists for RT-qPCR

Genes	Forward primer	Reverse primer
THY1	ATCGCTCTCCTGCTAACAGTC	CTCGTACTGGATGGGTGAACT
CTGF	CATCTCCACCCGGGTTACCAA	AGTACGGATGCACTTTTTGC
COL1A1	GAGGGCCAAGCACAAGACATC	CAGATCACGTCATCGCACAAC
TUJ1	GCGCATCAGCGTATACTACAA	TTCCAAGTCCACCAGAATGG
MAP2	TTGGTGCCGAGTGAGAAGAA	GGTCTGGCAGTGGTTGGTTAA
NURR1	GTTCAGGCGCAGTATGGGTC	CTCCCGAAGAGTGGTAACTGT
TH	CTGTGGCCTTTGAGGAGAAG	GGTGGATTTTGGCTTCAAAC
GAPDH	TGCACCACCAACTGCTTAGC	GGCATGGACTGTGGTCATGAG

### Electrophysiology analysis

Recording the electrophysiological properties of induced neurons was performed with the whole-cell patch-clamp techniques using an inverted microscope and an EPC-10 amplifier (HEKA). Induced neurons were maintained in external solutions consisting of 150 mM NaCl, 4 mmol/L KCl, 2 mmol/L CaCl_2_, 2 mmol/L MgCl_2_, 10 mmol/L glucose, and 10 mmol/L HEPES (pH 7.4, 300 mOsm). Patch pipettes were pulled to yield a resistance of 3–4 MΩ and filled with intracellular solutions that contained 130 mmol/L K-Gluconate, 3 mmol/L NaCl, 6 mmol/L KCl, 0.2 mmol/L EGTA, 4 mmol/L ATP-Mg, 0.4 mmol/L GTP-Na, 14 mmol/L phosphocreatine-di (Tris), and 10 mmol/L HEPES (pH 7.2, 285 mOsm). The pipette capacitance was neutralized, and access resistance was continuously monitored when recording. In the whole-cell current-clamp recordings, depolarizing step currents from − 60 pA to 120 pA were used to trigger action potentials at a 20 pA increment and 800 ms in duration. While in whole-cell voltage-clamp recordings, whole-cell currents were triggered with voltage steps ranging from − 60 to 30 mV at a 10 mV increment. 1 μmol/L tetrodotoxin (TTX) was added to the chamber, and the voltage steps were repeated to examine TTX-sensitive currents. Data analyses were performed with pClamp 9.0 (Axon Instruments).

### Statistical analysis

All statistical analyses were performed using GraphPad Prism (version 7, GraphPad Software, USA). Quantitative data were presented as mean ± SEM from three independent experiments. Statistical significance of differences between groups was determined by unpaired, two-tailed Student’s *t*-test and a *P*-value < 0.05 was considered significant.

## Results

### Phenotypical characterization of human fetal lung IMR-90 fibroblasts

Human fetal lung IMR-90 fibroblasts were routinely cultured in the DMEM medium containing 10% FBS. Microscopic images showed a spindle-shaped morphology (Fig. 1b). Immunostaining results showed that IMR-90 fibroblasts were positive for the fibroblast marker VIMENTIN (Fig. 1c). To determine whether IMR-90 fibroblasts were mixed with neuronal cells, we performed immunostaining assays for neural makers III β-tubulin (TUJ1) and microtubule-associated protein 2 (MAP2) in IMR-90 fibroblasts. As shown in Fig. 1c, IMR-90 fibroblasts did not express markers for neuronal cells. The absence of neural progenitor/stem cells (NPC/NSC), glial cells, and neural crest cells were determined by measuring the expression of NPC/NSC markers PAX6 (Paired box protein Pax-6), SOX2 (Sex-determining region Y-box 2), and NESTIN, glial cell marker GFAP, and neural crest cell marker p75. Immunostaining assay showed that cultured IMR-90 fibroblasts did not express PAX6, SOX2, NESTIN, GFAP, and p75, indicating no contamination of these neural cells (Fig. 1c).

### Conversion of IMR-90 fibroblasts into ihDAs using small molecules and protein factors

The small molecules and protein factors that are known to promote neural fate commitment and dopaminergic neuron development and differentiation were selected for the chemical induction. Before induction, IMR-90 fibroblasts were seeded on matrigel-coated plates or glass coverslips. After testing various combinations of small molecules and protein factors diluted in the neuronal induction medium (data not shown), we found that a combination of 0.1 mmol/L valproic acid (VPA), 2 μmol/L Respox (an inhibitor of transforming growth factor β, TGF-β signaling), 3 μmol/L kenpaullon (an inhibitor of glycogen synthase kinase-3β, GSK-3β), 5 μmol/L forskolin (adenylate cyclase activator), 5 μmol/L Y-27632 (an inhibitor of Rho-associated protein kinase, ROCK), 2 μmol/L purmorphamine (an activator of sonic hedgehog, SHH signaling), 100 ng/ml SHH, 100 ng/ml fibroblast growth factor-8b (FGF-8b), and 20 ng/ml basic fibroblast growth factor (bFGF), 50 ng/ml Wnt1, and 50 ng/ml Wnt5a could directly convert IMR-90 fibroblasts into neuron-like cells (Fig. 1a and b). After 6–8 days of culture in the neuronal induction medium, the cells demonstrated a morphology showing small, compact cell bodies and monopolar, bipolar, or multipolar projections (Fig. 1b). Afterward, the neuronal induction medium was replaced with the neuronal maturation medium and the culture continued for 7–14 days (Fig. 1a). After the maturation culture, the induced neurons showed complex morphologies with many dendrites and long axons (Fig. 1b).

**Fig. 2:**
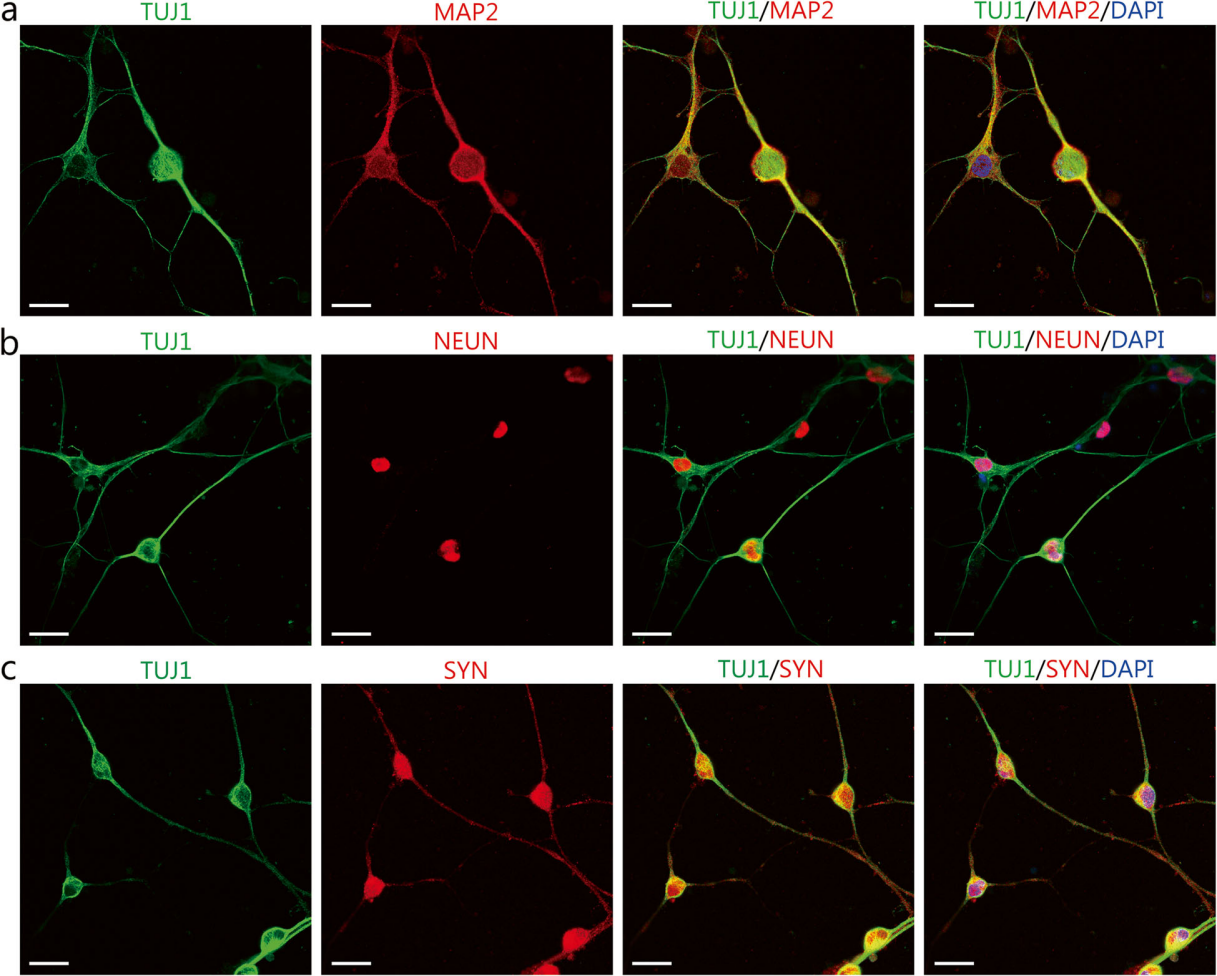
Phenotypic characterization of maturation neuronal markers in cells undergone the induction process. **a-c** Immunostaining assay for the mature neuron markers MAP2, neuronal nuclei (NEUN), and synapsin 1 (SYN) after 14 days of chemical induction. Scale bars = 25 μm

The immunostaining assays were conducted to characterize the molecular phenotypes of induced neuron-like cells. On day 7 after induction, the induced neuron-like cells were immunopositive for the pan-neuronal marker TUJ1 (Fig. 1d). Quantification showed that the percentage of TUJ1^+^ cells in the total DAPI^+^ cells was 94.74% ± 0.60%. The chemical induction induced a rapid downregulation of the fibroblast marker VIMENTIN and an upregulation of TUJ1 (Fig. 1e). In comparison, IMR-90 fibroblasts cultured in the control medium still expressed high levels of VIMENTIN and did not express TUJ1 (Fig. 1f). These data showed that chemical induction led to an increased expression of the neural markers and a decreased expression of the fibroblasts’ markers. After culturing in a mature neuronal medium, the induced neurons expressed the mature neuron markers MAP2 , neuronal nuclei (NEUN), and pre-synaptic marker Synapsin 1 (SYN), as shown in Fig. 2a-c.

**Fig. 3:**
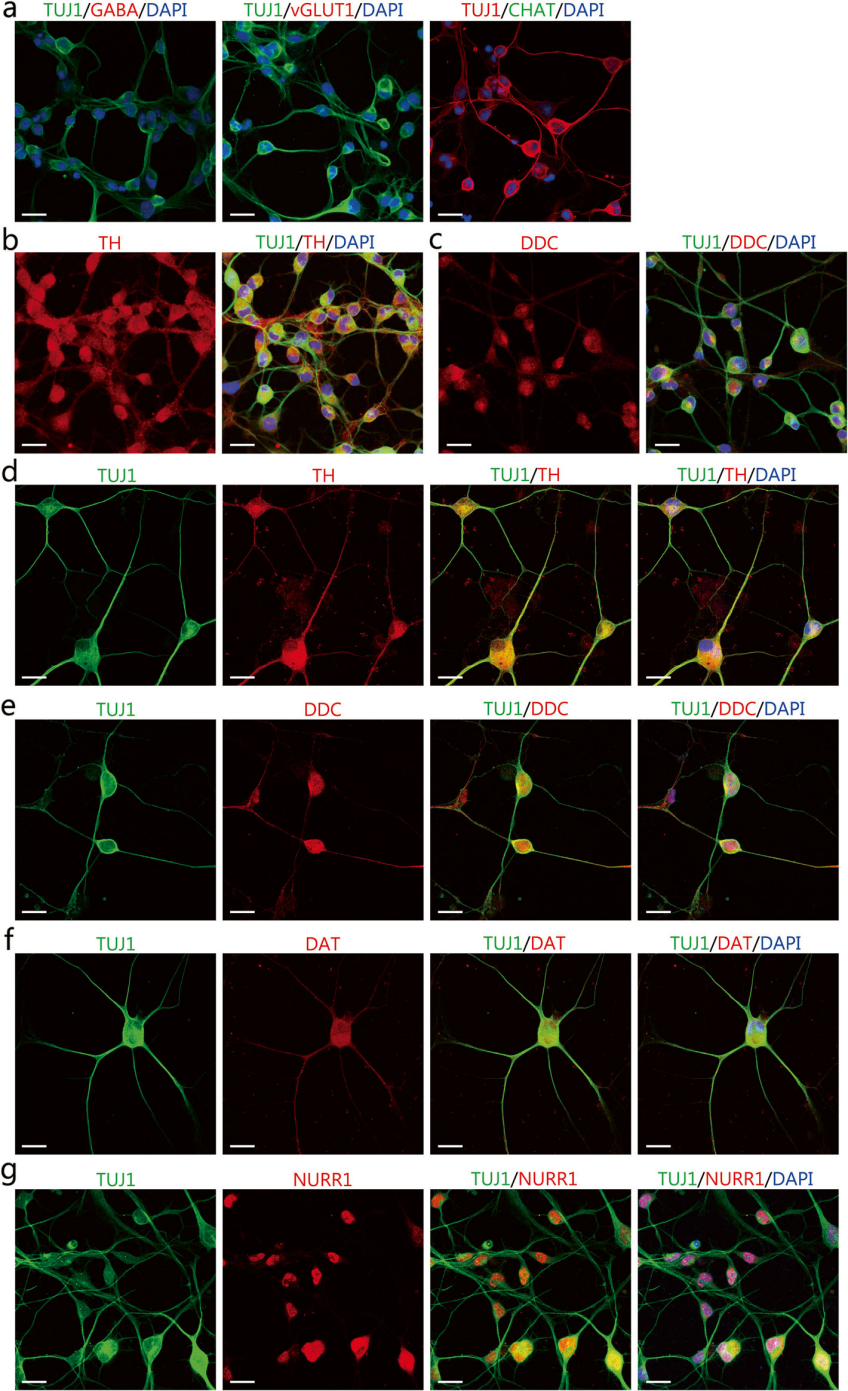
The expression of neuronal markers for subtype identification in the induced neuron-like cells. **a-c** Immunocytochemical analysis of induced neurons for the γ-aminobutyric acid (GABA), and vesicular glutamate transporter 1 (vGlut1), choline acetyltransferase (CHAT), tyrosine hydroxylase (TH), and dopa decarboxylase (DDC) in induced neurons after 4 days of chemical induction. Scale bars = 25 μm. **d-g** Immunocytochemical analysis of the expression of TH, DDC, DAT, and NURR1 in induced neurons after 14 days of chemical induction. Scale bars = 25 μm

To determine the neuronal subtype of the induced neurons, we examined the expression of the GABAergic neuron marker gamma-aminobutyric acid (GABA), glutamatergic neuron marker vesicular glutamate transporter 1 (vGlut1), and cholinergic neuron marker choline acetyltransferase (CHAT), and DA neuron marker tyrosine hydroxylase (TH). On day 4 of the chemical induction, the induced neurons were negative for GABA, vGlut1and CHAT, but positive for TH (Fig. 3a and b). Moreover, the induced neurons also expressed another DA neuron marker dopa decarboxylase (DDC, Fig. 3c). Therefore, the induced neurons may be DA neuron-like cells. On day 14 of the chemical induction, the induced neurons expressed DA neuron markers TH, DDC, dopamine transporter (DAT), and nuclear receptor-related 1 (NURR1), an orphan nuclear receptor expressed by DA neurons (Fig. 3d-g). At this time point after the maturation culture, we further determined the TUJ1^+^TH^+^ cell yields relative to the total DAPI^+^ cells and the purity of TUJ1^+^TH^+^ cells relative to TUJ1^+^ neurons. Quantitative analysis showed that 87.88% ± 2.03% of the total DAPI^+^ cells were both positive for TUJ1 and TH, indicating high DA neuron-like cell yields after the chemical conversion. The percentage of TUJ1^+^TH^+^ cells within the induced TUJ1^+^ neurons was 92.79% ± 1.71%. This result indicated high purity of TH^+^ DA neurons among the induced neurons from fibroblasts. Thus, the chemical method induced IMR-90 fibroblasts into ihDAs with high cell yields and purity.

### Gene expression analysis of ihDAs

**Fig. 4:**
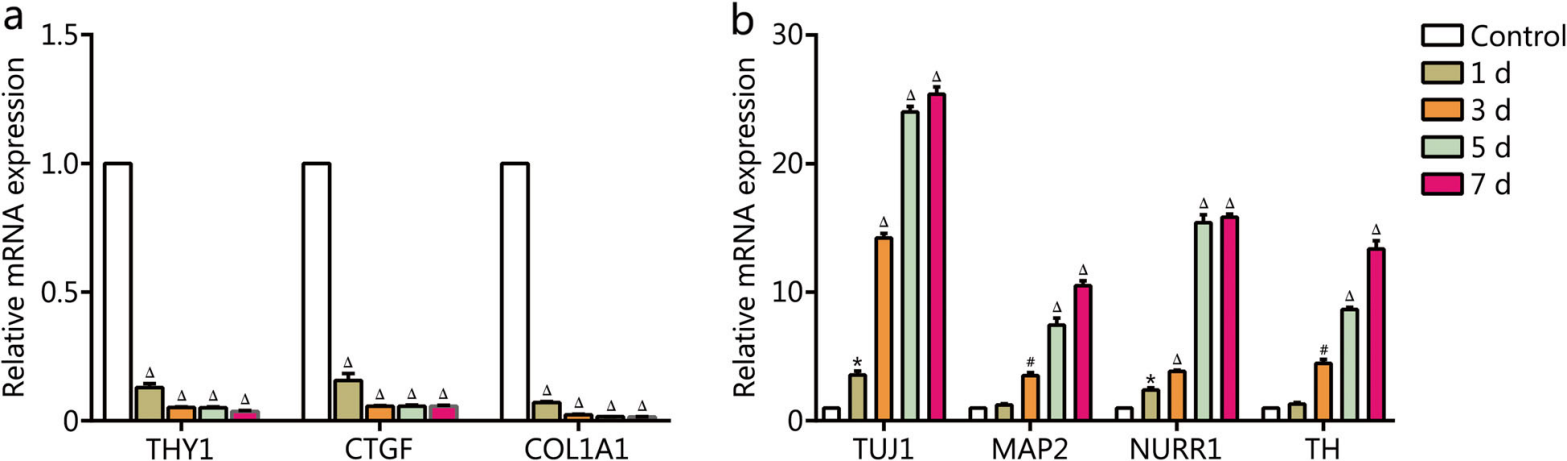
The gene expression analysis during the chemical induction. RT-qPCR analysis of mRNA expression levels of fibroblast genes *THY1, CTGF, and COL1A1*, neuron-associated genes *TUJ1* and *MAP2*, and DA neuron-associated genes *TH* and *NURR1* during the chemical induction. Values are presented as mean ± SEM (*n* = 3. *P < 0.05, #*P* < 0.001, △*P* < 0.0005, versus control (day 0)

The gene expression of ihDAs acquired after the chemical induction were analyzed by RT-qPCR. The results showed that the chemical induction led to a significant downregulation of the fibroblast marker genes *THY1*, *CTGF*, and *COL1A1* (Fig. 4a) and a significant upregulation of neural genes *TUJ*1 and *MAP2* and DA neuron-associated genes *TH* and *NURR1* (Fig. 4b). The alterations in gene expression suggested that the conversion process was accompanied by inhibition of the fibroblast-specific genes and activation of the neuronal genes.

### The conversion of IMR-90 fibroblasts into ihDAs did not pass through an NPC/NSC intermediate stage

**Fig. 5:**
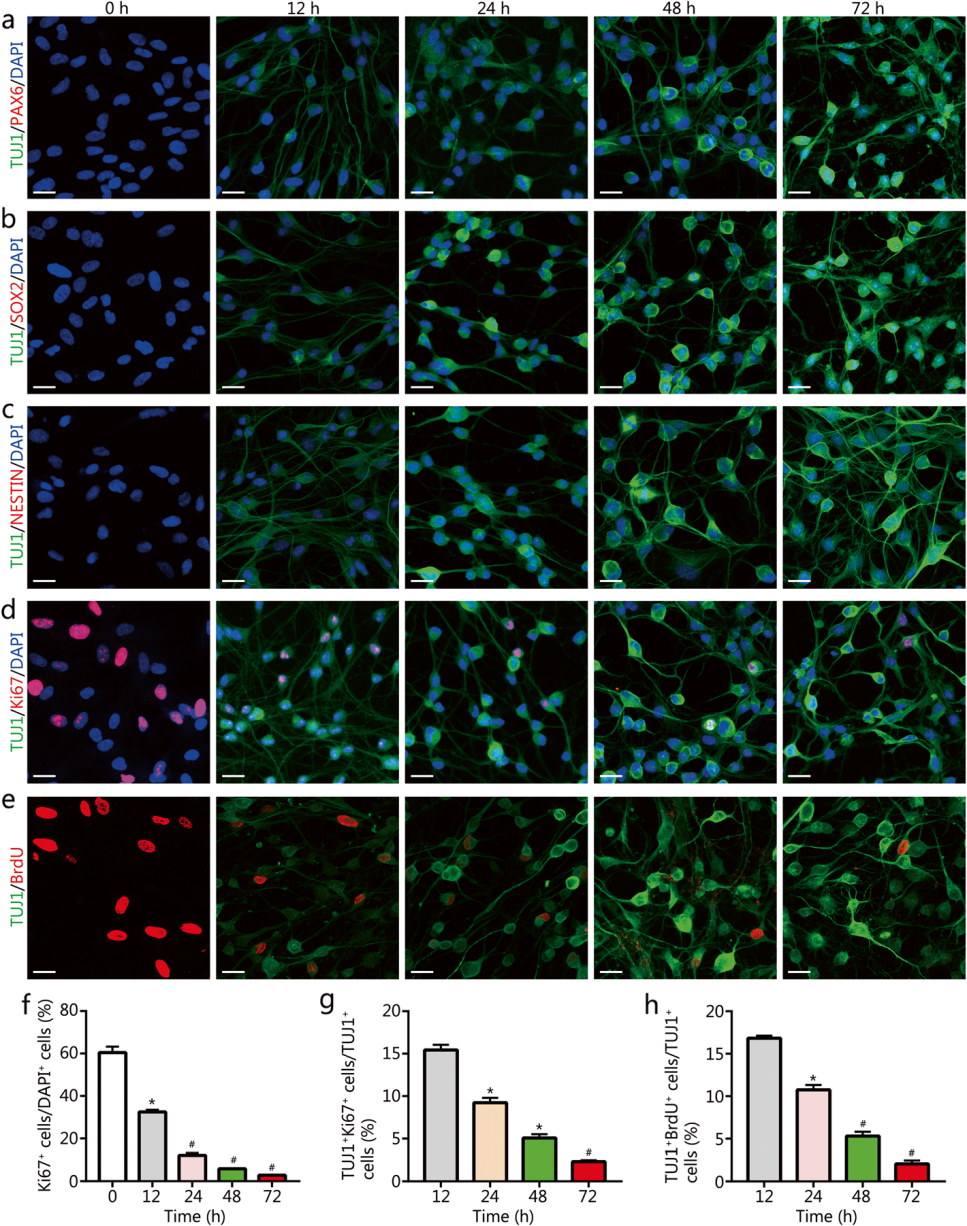
The expression of NPC/NSC and proliferative markers in the ihDAs. **a-c** Immunostaining analysis of expression of NPC/NSC markers PAX6, SOX2, and NESTIN in IMR-90 fibroblasts after 0–72 h of chemical induction. Scale bars = 25 μm. **d** and **e** Immunostaining analysis of proliferation markers Ki67 and BrdU after 0–72 h of chemical induction. Sale bars = 25 μm. **f** Quantification of the percentage of Ki67^+^ cells relative to the total DAPI^+^ cells (mean ± SEM from 10 randomly selected 20× visual fields from triplicate samples), *P < 0.001, #*P* < 0.0005, versus control (0 h). **g** Quantification of the percentage of TUJ1^+^Ki67^+^ cells relative to TUJ1^+^ cells at 12, 24, 48, and 72 h after chemical induction (mean ± SEM from 10 randomly selected 20× fields from triplicate samples), *P < 0.001, #*P* < 0.0005, versus 12 h. **h** Quantification of the percentage of TUJ1^+^BrdU^+^ cells relative to TUJ1^+^ cells at 12, 24, 48, and 72 h after chemical induction (mean ± SEM from 10 randomly selected 20× visual fields from triplicate samples), *P < 0.001, #*P* < 0.0005, versus 12 h

We further examined whether the neuronal conversion process involved the formation of the NPC/NSC intermediate state. Immunostaining assays demonstrated no expression of NPC/NSC markers PAX6, SOX2, and NESTIN during the early conversion stage from 0 to 72 h (Fig. 5a-c). These data indicated no formation of NPC/NSC. Moreover, immunostaining assays showed a rapid decrease in the number of cells stained with the proliferation marker Ki67 after induction (Fig. 5d and f). The percentage of TUJ1^+^Ki67^+^ cells relative to TUJ1^+^ cells decreased over the chemical process, and at 72 h after induction, less than 3% of TUJ1^+^ cells were co-stained with Ki67 (Fig. 5g), suggesting no cell expansion during the chemical induction. Proliferating cells were pulse-labeled with 5-bromo-2-deoxyuridine (BrdU) for 2 h before immunostaining at 0, 12, 24, 48, and 72 h after the chemical induction, respectively. The BrdU-labeled cells seemed to decline over the chemical induction process, and only about 2% of TUJ1^+^ cells incorporated BrdU at 72 h after chemical induction (Fig. 5e and h). These data together suggested that the conversion process did not pass through an NPC/NSC intermediate stage, accompanied by loss of proliferation.

### The ihDAs possess the electrophysiological characteristics of neurons

**Fig. 6:**
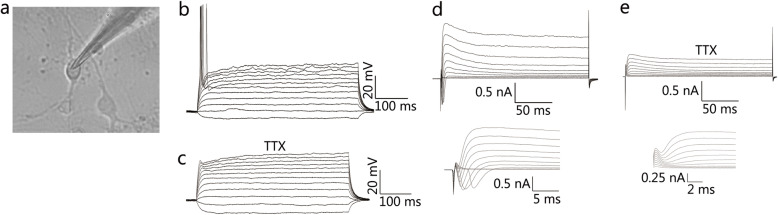
The analysis of electrophysiological properties of ihDAs. **a** Representative image of the ihDAs by whole-cell patch-clamp recording. **b** Current-clamp recordings of ihDAs, showing single action potential (*n* = 5/10, recorded cells). **c** Tetrodotoxin (TTX) could inhibit action potentials. **d** In voltage-clamp mode, representative traces of whole-cell currents, with inward sodium current and outward potassium current (*n* = 7/10, recorded cells). **e** Treatment with TTX could block the inward sodium current

Since neurons possess electrophysiological properties, we then investigated the electrophysiological activities on ihDAs with the whole-cell patch-clamp technique on 14–21 days after induction (Fig. 6a). In the whole-cell current-clamp mode, ihDAs were found to fire single action potentials after triggering with depolarizing current steps (*n* = 5/10, recorded cells, Fig. 6b). In the whole-cell voltage-clamp mode, depolarizing voltage steps triggered a rapid inward current and slow outward currents (Fig. 6d), suggesting an opening of voltage-activated sodium channels and potassium channels, respectively. The sodium channel blocker tetrodotoxin (TTX) could block the inward current (Fig. 6e) and action potentials (Fig. 6c). Taken together, these results suggested that ihDAs might possess the electrophysiological characteristics of neurons.

## Discussion

Previous studies have demonstrated that the mouse and human fibroblasts could be converted into neurons, including dopaminergic neurons, by ectopic expression of various combinations of specific transcription factors [11, 13–16, 20–22]. However, such approach is associated with safety concerns due to the genetic manipulation. In the current study, we identified that a combination of small molecules and growth factors could effectively induce IMR-90 fibroblasts into dopaminergic neuron-like cells that expressed dopaminergic neuron markers and possessed the electrophysiological properties of neurons.

Our induction method is based on the theory that modulation of the signaling pathways responsible for neuron development and differentiation can induce somatic cells into neuron fates. Previous studies have shown that VPA, CHIR99021 (an inhibitor of GSK-3β), and Repsox (an inhibitor of TGF-β signaling) could induce NPCs from mouse and human fibroblasts [23]. Among them, inhibitors of GSK-3β and TGF-β signaling have been demonstrated to increase the neuronal conversion from fibroblasts in the presence of two transcription factors Ascl1 and Ngn2 [24]. Both studies indicated the possibility to induce fibroblasts directly into neuron-like cells if providing additional factors that promote neuronal differentiation. Additionally, forskolin and Y-27632 (an inhibitor of ROCK signaling), two small molecules that promote neuronal differentiation, has been demonstrated to induce fibroblasts into neuron-like cells [18]. In line with this, the combination of VPA, Repsox, kenpaullone (another inhibitor of GSK-3β), forskolin, and Y-27632 could induce human IMR-90 fibroblasts into neuron-like cells. Furthermore, we selected additional factors that can induce DA neuron cell fate. As activation of SHH signaling is required for DA neuron development and differentiation, SHH protein and small molecule purmorphamine that activate SHH signaling have been commonly used to induce DA neuron differentiation from embryonic stem cells and induced pluripotent stem cells [25–27]. The cocktail also included FGF-8b, bFGF, Wnt1 and Wnt5a, which are essential for DA neuron development, neurogenesis and survival [25, 28–33]. These small molecules and protein factors may synergistically direct the conversion of human fibroblasts into ihDAs.

The ihDAs expressed the pan-neural markers TUJ1, MAP2, NEUN, SYN and DA markers TH, DDC, DAT, and NURR1, evidenced by immunostaining assays. The protein NURR1 is a later transcription factor responsible for DA maturation and maintenance of function [25, 34–37], which was expressed in the ihDAs. The RT-qPCR analysis revealed a significant downregulation of fibroblast-specific genes *THY1*, *CTGF*, and *COL1A1* and an upregulation of neuronal genes *TUJ1* and *MAP2* and DA-specific genes *TH* and *NURR1*. Pfisterer et al. [14] reprogrammed human embryonic fibroblasts to DA neurons using five transcription factors ASCL1, LMX1A, BRN2, MYT1L, and FOXA2, with a conversion efficiency of approximately 20% TH^+^ cells. The induced DA neurons expressed the TUJ1, TH, DDC, and NURR1, evidenced by immuocytometry analysis. Caiazzo et al. generated DA neurons from IMR-90 fibroblasts by ectopic expression of ASCL1, NURR1, and LMX1A and found that cell numbers of TUJ1^+^ cells and TH^+^ cells were 10% ± 4 and 6% ± 2% of the infected cells, respectively. The same three transcription factors also converted adult human fibroblasts to DA neurons with a lower efficiency of 5% ± 1% TUJ1^+^ cell yields and 3% ± 1% TH^+^ cell yields. The induced DA neurons expressed TUJ1, TH, DDC, DAT, VMAT2, and ALDH1A1 profiled by immunostaining assay and RT-PCR analysis [15]. Liu and colleagues reported an alternative combination of transcription factors ASCL1, NGN2, SOX2, NURR1, and PITX3 that could convert human IMR-90 fibroblasts into DA neuron-like cells, in which approximately 40% of the total DAPI-stained cells were positive for DDC, the DA neuron-specific marker [16]. In addition, immunostaining assays showed the expression of TUJ, TH, and DAT in the induced neurons, and RT-qPCR also revealed an increase in the mRNA expression levels of DA-specific genes *TH*, *DAT*, *VMAT2*, and *EN1* in induced DA neuron-like cells [16, 38]. Comparing to the above studies, ihDAs induced by our method shared similar phenotypic characteristics with other induced human DA neurons by transcription factors. Interestingly, the cocktail in our study had a higher neuronal conversion efficiency and a higher purity of TH^+^ cells.

Functionally, ihDAs possess the electrophysiological properties of neurons, with about 50% of the recorded neurons capable of firing single action potentials and triggering inward sodium currents and outward potassium currents, which was consistent with the ability of the induced DA neurons to fire single action potential in Liu’s and Caiazzo’s study [15, 39]. Whereas the induced DA neurons from Pfisterer’s group and Jiang’s group were found to fire single and multiple action potentials [14, 39], suggesting that the induced neurons may possess more mature membrane properties. It is known that co-culture of glia and neurons could promote the membrane maturation of neurons. Since the ihDAs were not cultured on glia monolayer in the current study, this might partially explain why the cells displayed immature membrane properties and could not fire multiple action potentials. Whether the ihDAs could fire multiple action potentials after a longer-term of culture remains to be explored. Nevertheless, the ihDAs had the basic electrophysiological features of neurons.

Moreover, the conversion process did not pass through an NPC/NSC intermediate state, with no detection of NPC/NSC markers and a significant loss of proliferation after the induction, which appeared to diverge from the embryonic development process of DA neurons. How this difference might impact the function of ihDAs needs to be clarified in the transplantation studies. Overall, these data consistently showed that ihDAs from human fibroblasts by the chemical cocktail shared some similar characteristics of DA neurons. Whether the induced ihDAs could maintain the phenotype of DA neurons after transplantation in vivo and function as normal DA neurons needs to be evaluated in the future work.

## Conclusions

In this study, we develop a chemical cocktail comprising small molecules and specific protein factors to produce DA neuron-like cells rapidly and efficiently from human fibroblasts, without transfection of exogenous genes. The strategy may provide a potential cellular source for cell-based therapy of Parkinson’s disease. However, it should be further evaluated whether the induced neurons are functional in vivo.

## Data Availability

The data and materials used in the current study are all available from the corresponding author upon reasonable request.

## Abbreviations

DA: Dopaminergic
NPC: Neural progenitor cell
NSC: Neural stem cell
MAP2: Microtubule-associated protein 2
TUJ1: III β-tubulin
RT-qPCR: Quantitative real-time polymerase chain reaction
TTX: Tetrodotoxin
